# On effectively predicting autism spectrum disorder therapy using an ensemble of classifiers

**DOI:** 10.1038/s41598-023-46379-3

**Published:** 2023-11-15

**Authors:** Bhekisipho Twala, Eamon Molloy

**Affiliations:** 1 Office of the Deputy Vice-Chancellor (Digital Transformation), Tshwane University of Technology, Private Bag x680, Pretoria, 001 South Africa; 2 Waterford Institute of Technology, School of Science & Computing, Waterford, Ireland

**Keywords:** Computational biology and bioinformatics, Health care, Neurology, Mathematics and computing

## Abstract

An ensemble of classifiers combines several single classifiers to deliver a final prediction or classification decision. An increasingly provoking question is whether such an ensemble can outperform the single best classifier. If so, what form of ensemble learning system (also known as multiple classifier learning systems) yields the most significant benefits in the size or diversity of the ensemble? In this paper, the ability of ensemble learning to predict and identify factors that influence or contribute to autism spectrum disorder therapy (ASDT) for intervention purposes is investigated. Given that most interventions are typically short-term in nature, henceforth, developing a robotic system that will provide the best outcome and measurement of ASDT therapy has never been so critical. In this paper, the performance of five single classifiers against several multiple classifier learning systems in exploring and predicting ASDT is investigated using a dataset of behavioural data and robot-enhanced therapy against standard human treatment based on 3000 sessions and 300 h, recorded from 61 autistic children. Experimental results show statistically significant differences in performance among the single classifiers for ASDT prediction with decision trees as the more accurate classifier. The results further show multiple classifier learning systems (MCLS) achieving better performance for ASDT prediction (especially those ensembles with three core classifiers). Additionally, the results show bagging and boosting ensemble learning as robust when predicting ASDT with multi-stage design as the most dominant architecture. It also appears that eye contact and social interaction are the most critical contributing factors to the ASDT problem among children.

## Introduction

World autism awareness month is celebrated in April worldwide by people and organisations alike. This entire month is dedicated to raising awareness, sharing understanding, and shedding light on a global and South African health crisis, which parents have been battling for years. Autism Spectrum Disorder (ASD) can be defined as a developmental disability that affects social interaction, communication and learning skills (where the spectrum reflects a wide range of symptoms that the child can present). Autism therapies are interventions that attempt to lessen the deficits and problem behaviours associated with ASD.

About 1 in 160 children worldwide are diagnosed with ASD, with a higher rate of 1 in 68 children in the United States (Centers for Disease Control^1^). Abnormalities characterise themselves in social interactions and patterns of communication and a restricted, stereotyped, repetitive repertoire of interests and activities (WHO^2^). However, the treatment and intervention services for ASD are tricky since there are time-consuming treatments conducted. The ASD symptoms typically appear in the first 2 years of a child’s life, developing in a specific period. There are many types of treatments available including behavioural, developmental, educational, social-relational, pharmacological and psychological. One South African group, Quest School (Sowetan LIVE^3^) has proposed that children be diagnosed when they are young, not at 5 years of age as is the norm.

We cannot overemphasise the impact ASD has on adults and children. The common signs in adults include finding it hard to understand what others are thinking or feeling and getting very anxious about social situations. For young children it includes not responding to their name, avoiding eye contact, and repetitive movements such as flapping their hands and flicking their fingers or rocking their bodies. Signs of ASD for older children include not seeming to understand what others are thinking or feeling, unusual speech, such as repeating phrases and talking “at” others or finding it hard to say how they feel. ASD can sometimes be different in girls (women) and boys (mean) with it being harder to spot especially in girls (women). Women may have learnt to hide signs of ASD to “fit in” by coping with people who do not have ASD while girls may hide some signs of ASD by copying how other children behave and play^4^.

Recently, there has been an explosion in ASD cases worldwide, and they have been increasing at an alarming rate (Centres for Disease and Control^1^). The World Health Organisation (WHO^5^; Wolff and Piven^6,7^ have argued that 1 out of every 160 children has ASD. A certain percentage of people with this disorder have been shown to live independently, whilst others would require life-long care and support. ASD traits are difficult to trace due to tests and diagnoses requiring significant time and cost. However, several treatments, therapies and interventions can help children with ASD improve their abilities and reduce their symptoms.

The topic of ASD therapy enhancement has been of interest to researchers for decades as the effects of a robot-enhanced intervention for children with ASD. There has been a lot of research work in the spheres of machine learning (ML) and statistical pattern recognition (SPC), where communities were discussing how to combine models or model predictions^8^. Furthermore, much research work in these communities has shown that an ensemble learning of classifiers is an effective technique for improving predictive accuracy (due to its variance reduction benefit). Ensemble learning of classifiers’ development and successful fielding have significantly lagged behind bio-medical and health science research activities, yet it has been prominent in other fields. A central concern of these applications is the need to increase the predictive accuracy of early ASD diagnosis and test decisions.

The basic idea behind ensemble learning is to train multiple classifier learning systems to achieve the same objective and then combine their predictions. There are different ways ensembles can be developed and the resulting output combined to classify new instances. The popular approaches to creating ensembles include changing the cases used for training through techniques such as bagging^9^, boosting^10^, stacking^11^, changing the features used in training^12^, and introducing randomness in the classifier itself^13^.

Due to the nature of ASD and its impact on societies, an improvement in predictive therapy enhancement accuracy or even a fraction of a per cent translates into significant future savings in time, costs, and even deaths^14–16^. Furthermore, the economic effect of ASD on individuals with the disorder, their families, and society as a whole has been poorly understood and has not been updated in light of recent ASD prediction and detection findings. This enormous effect on families warrants better therapeutic, prediction and detection methods from machine learning and statistical pattern recognition communities.

Supporting the development of a child with ASD is a multi-profile therapeutic work on disturbed areas, especially understanding and linguistic expression used in social communication, development of mutual social contacts and functional or symbolic play. In recent years, robot learning^17^ and robot-assisted ASD therapy (RET) have grown in popularity. The key research findings on RET have shown its effectiveness for children with ASD in particular: communication (common attention, imitation, undertaking communication behaviours, recognizing and understanding emotions and developing sensitivity to physical contact^18–22^, Chernyak et al.^23^).

Other tools that are being embraced by therapists, counsellors, teachers, parents and their children to help those with ASD to better communicate and connect with others are virtual reality (VR) and augmented reality (AR). Several research studies examined suggest promising findings about the effectiveness of virtual and augmented reality-based treatments for the promotion, support, and protection of health and well-being in children and adolescents with ASD. VR and AR have also been used to help those without ASD understand what living with the condition means^24–26^.

Using a variety of machine learning techniques, one could analyze the parent’s age, socio-economic status and medications to predict a child’s ASD diagnosis. Predictive algorithms could also be useful for identifying factors that may contribute to ASD. For example, machine learning algorithms helped find an association between ASD and a parent’s use of substances such as caffeine and certain antidepressants^27^. Machine learning has also been used to better understand (or classify) why ASD traits vary in their nature and severity from person to person^28^. This was after another machine learning study by Stevens et al.^29^ analyzed behavioural data and found two overarching behavioural profiles of ASD, each with its subgroups based on the severity of different traits. Other scholars have investigated the clinical applications of robots in the diagnosis and treatment of ASD (Diehl et al.^30^) while others have created machine-learning algorithms that could help robots understand when an autistic child needs help^31,32^.

Several other studies for predicting ASD traits in an individual have been carried out by the ASD research and data (science) analytics community using several machine learning (ML) and statistical modelling techniques. These include screening detection, identification, classification and prediction of ASD traits in an individual.

For screening detection, alternating and functional decision trees^33–35^, support vector machines^36^ and “red flags”^37^ have been used while support vector machines have been used for both detection and identification^38^. Kosmicki et al.^33^ investigated logistic model trees and logistic regression for detecting non-ASD against ASD among children.

To predict ASD traits, a support vector machine, a naïve Bayes classifier, and the random forest have been further applied by^39^. Prediction of ASDT response from baselines fMRI using random forests and tree bagging was proposed by Dvornek et al.^40^ with their learning pipeline method achieving higher accuracy compared with standard methods. Bala et al.^41^ investigated the identification of ASD among toddlers, children, adolescents and adults using several machine learning algorithms (K-Star, classification and regression trees, *k*-nearest neighbour, support vector machine, bagging and random tree). SVM achieved the best performance for the prediction of ASD at different age levels.

Deep learning and neural networks have been used by Heinsfeld et al.^38^ to predict ASD patients using imaging of the brain with a follow-up research work on classification and hemodynamic fluctuations by Xu et al.^42^. An empirical comparison of Adaboost, flexible discriminant analysis (FDA), decision tree (C5.0), boosted generalised linear model (GLMboost), linear discriminant analysis (LDA), mixture discriminant analysis (MDA), penalised discriminant analysis (PDA), support vector machines (SVM) and classification and regression trees (CART) for early stage detection of ASD for toddlers, children and adults. Good performances were observed for SVM (toddlers), Adaboost (children) and GLMboost (adults)^43^.

Recently, Kanchana et al.^44^ predicted early phases of ASD in adults using naïve Bayes, logistic regression, random forest and random tree with random forest achieving the highest predictive accuracy.

From the review, it is evident that all the researchers have used single classifiers for detecting, predicting or classifying ASD in general yet ensemble models have been more stable and, more importantly, shown to predict better than single classifiers. They have also been known to reduce model bias and variance. Some studies have assessed the ethical and social implications of translating embodied artificial intelligence (AI) applications into mental health care across the fields of Psychiatry, Psychology and Psychotherapy^45,46^. Furthermore, despite the limitations of single classifiers which include them not being able to make predictions on new data that they have not seen before because all the historical information must be provided in advance and the overfitting problem (i.e. overfitting the data they are looking at).

None of the research studies have looked at predicting ASD therapy (ASDT) and ways to improve ASDT predictive accuracy. Yet, proactive corrective actions can be taken well in advance if the prediction of ASDT is even more accurate. A slight increase in therapy predictive accuracy will not only have a positive impact on foreseeing ASD in toddlers but also improve outcomes for children with ASD thereby reducing symptoms due to early behavioural interventions.

This research work proposes modelling using an ensemble of classifiers approach to help show their effectiveness when predicting ASDT in terms of a robot-assisted intervention group (Robot-Enhanced-Therapy) and a control group receiving intervention by humans only (Standard-Human-Treatment) conditions, respectively. The investigation aims to find out if the use of artificial intelligence algorithms can help identify a reliable method for identifying ASD children most likely to benefit from a specific intervention program in advance and a solid foundation for establishing a personalised intervention program recommendation system for ASD children.

Such an ensemble learning approach is used to overcome precariousness in predictions and to enhance the accuracy and efficiency of the predictions. The MCL systems architecture and resampling processes are also considered. In other words, this research work focuses on predicting the effectiveness of ASD-enhanced therapy using a social robot and a human being.

To this end:

1. The first significant contribution of the paper is the investigation of five single-classifier learning systems to identify the best performing in terms of predicting therapy enhancement for autistic children using a social robot the type of therapy (on the one hand) and standard human treatment (on the other hand).
2. The second contribution is the proposal of a multiple-classifier learning system (or ensemble learning) approach to predict ASD therapy enhancement. The idea is to assess if using such a multiple classifier learning systems (MCLS) approach will be worthwhile to overcome the limitations of a single classifier learning system (SCLS) in terms of predictive accuracy due to their inability to handle more complex tracking situations with high accuracies. To analyse the performance of MCLS over SCLS, the unique models must be accurate individually and they need to be sufficiently diverse. For this reason, all possible combinations of the number of classifiers per ensemble are explored (i.e. from two classifiers per ensemble to five classifiers).
3. Finally, feature selection through a decision tree-based approach is used to identify which physical characteristics are most significant in ASDT treatment.

To the best of our knowledge, this study is one of the few if not the first study that investigates the application of artificial intelligence in ASDT.

The rest of the paper is organised as follows: Sect. “Single-classifier learning systems” gives a background on single-classifier learning systems used for ASDT prediction. Then multi-classifier learning systems are examined from the intelligibility viewpoint to improve the effectiveness of ASDT predictive accuracy (Sect. “Multiple classifier learning systems”). Section “Experiments” presents the experimental design in set-up and results drawn from a DREAM dataset, supporting a data-driven study of ASD and robot-enhanced therapy. Finally, the paper is concluded with critical research findings and remarks in Sect. “Remarks and conclusion”.

## Single-classifier learning systems

There are several approaches to single-classifier learning. However, only five base methods of classifier construction are considered in this paper (i.e. a mixture of regression and tree-based, nets, instance-based and Bayesian-related). These include artificial neural network (ANN), decision tree (DT), *k*-nearest neighbour (*k*-NN), logistic discrimination (LgD) and the Naïve Bayes classifier (NBC). A brief description of the five classifiers and their use for classification or prediction tasks is now given.

### Logistic discrimination

Logistic discrimination (LgD) is a supervised learning classification algorithm used to predict the probability of the target variable (for example, a class). It was initially developed by Cox^47^ and later modified by Day and Kerridge^48^. LgD is related to logistical regression due to the dependent variable being dichotomous. In other words, only two possible values can be taken (for example, either 0 for non-detection of ASD or 1 for detecting ASD). For LgD, the probability density functions for the classes are not modelled like most supervised Learning Classification Methods but rather the ratios between them (i.e. it is partially parametric).

An unknown instance is a new element classified using a cut-off point score where the error rate is lowest for the cut-off point = 0.5^49^. The slope of the cumulative logistic probability function is steepest $\pi_{i} = 0.5$ $\pi_{i} \ge 0.5$ $\pi_{i} < 0.5$ at the halfway point [i.e. the logit function transforms continuous values to the range (0, 1)], which is necessary since probabilities must be between 0 and 1. The LgD approach can be generalised to more than two classes (also called multinomial logit models). Multinomial Logit Models (MLMs) are derived similarly to the LgD models. For more details about MLMs and other modified versins of LgD, the interested reader is referred to Jolliffe^50^ and, Hosmer and Lameshow^51^ referred to the interested reader.

### k-nearest neighbour

The *k*-nearest neighbour (*k*-NN) or instance-based learning approach is one of the most venerable and easy-to-implement machine learning algorithms for supervised and sometimes unsupervised learning^52^. *k*-NN can solve both classification and regression (prediction) problems by assuming that similar things exist near each other. Thus, the *k*-NN hinges on this assumption being true enough for the algorithm to be valid. Essentially, *k*-NN works by assigning the classification (or regression prediction) of the nearest set of previously classified (predicted) occurrences to an unknown instance. The memory is the storage for the entire training set.

To classify a new example, a distance measure (such as Hamming, Cosine similarity, Chebychev, Euclidean, Manhattan or Minkowski) is computed between the trained and unknown instances. For this paper, the Cosine similarity distance measure is used. Each stored training and the unknown instance are assigned the class of that nearest neighbouring instance. These Nearest Neighbours are first computed, and then the new example is given the most frequent class among the *k* neighbours. To select the value of* k* that is right for your data, the algorithm is run several times with different values of *k*. It reduces the number of errors encountered while maintaining the algorithms’ ability to make predictions when given data not seen before accurately. For the paper, the process of supervised learning will be focused on.

### Artificial neural networks

Like most state-of-the-art classification methods, Neural Networks or artificial neural networks (ANNs) are non-parametric (i.e. no assumptions about the data are made, as is the case with models such as linear regression). Instead, they are computational models inspired by an animal’s nervous system. These are represented by connections (layers) between many simple computing processors or elements (“neurons”). They have been used for various classification and regression problems in economics, forensics, and pattern recognition^53^. The ANN is trained by supplying it with many numerical observations or the patterns to be trained (input data pattern) whose corresponding classifications (desired output) are known. The final sum-of-squares error (SSE) over the validation data for the network is calculated when training the network. This SSE value is then used to select the optimum number of hidden nodes resulting in a trained neural network.

A new unknown instance is carried out by sending its attribute values to the network’s input nodes, where weights are applied to those values. Finally, the values of the output unit activations are computed. The weights and biases can be optimised by running the network multiple times. Its most significant output unit activation determines the classification of the new instance.

### Decision trees

A Decision Tree (DT) classifier is a supervised machine learning algorithm used for regression and classification tasks. It starts with a single node (subsequently, a series of decisions) and branches into possible outcomes, giving it a tree-like diagram^54,55^. When training a DT, the best attribute is selected (using the information gain measure) from the total attributes list of the data for the root node, internal node and leaf or terminal nodes. A DT classifier is simple to understand, interpret and visualise.

According to Safavian and Landgrebe^56^, a DT classifier has four primary objectives. These are (1) Classifying the training sample correctly as much as possible, (2) Generalising beyond the training sample so that unseen samples could be classified with high accuracy; (3) quickly updating the DT as more training samples become available (which is similar to incremental learning), and (4) Having a simple DT structure as possible. Despite the DT classifier strengths, Objective (1) is highly debatable and, to some extent, conflicts with Objective (2). Also, not all DT classifiers are concerned with objective (3). DTs are non-parametric and valuable to represent the logic embodied in software routines.

A DT takes as input a case or example described by a set of attribute values and outputs a Boolean multi-valued “decision,” making it easy to build automated predictive models. For this paper, the Boolean case is considered. Classifying an unknown instance is easy once the tree has been constructed. Starting from the root node of the DT and applying certain test conditions would eventually lead you to a leaf node with a class label associated with it. The class label associated with the leaf or terminal node is assigned to the instance.

### Naïve Bayes classifier

The Naïve Bayes classifier (NBC) is perhaps the most superficial and widely studied supervised probabilistic machine learning (ML) method that uses Bayes’ theorem with strong independence assumptions between the features to procure results. The NBC assumes that each input attribute variable is independent of training the data. This can be considered a naïve assumption about real-world data. Then, the conditional probability of each attribute *A*_*i*_*,* given the class label *C* is learnt from the training data^57,58^.

The strength of the NBC lies in its ability to handle an arbitrary number of independent numerical or categorical attribute features. The solid but often controversial primary assumption (due to its “naivety”) is that all the attributes *A*_*i*_ are independent given the value of class *C*. For classification, the Bayes rule is applied to determine the class of the unknown instances by computing the probability of *C* given ${A}_{1},\dots ,{ A}_{n}$ and then selecting the class with the highest posterior probability. The “naive” assumption of conditional independence of a collection of random variables is very important for the above result. Otherwise, it would be impossible to estimate all the parameters without such an assumption. This is a relatively strong assumption that is often not applicable. However, any bias in estimating probabilities may not make a difference in practice – it is the order of the probabilities and not the exact values that determine the probabilities.

Nonetheless, NBC has been shown to solve many complex real-world problems and to do so effectively. Also, it requires a small amount of training data to estimate the parameter. A frequency table is created for each attribute against the target (class) to calculate the posterior probability of classifying an unknown instance. Then, the NBC is used to calculate the posterior distribution. Once again, the prediction outcome is the class with the highest posterior probability.

## Multiple classifier learning systems

A multiple-classifier learning system (MCLS) can be defined as a set of classifiers whose individual predictions are combined in some way to classify new examples to produce one optimal predictive model. The most common type of MCLS includes an ensemble of classifiers that function for a parallel classifier input combination. Furthermore, a significant number of methods have been used to create and combine such individual classifiers, including ensemble methods, committee, classifier fusion, combination, aggregation, etc.

Once an MCLS is built and an aggregation determined, one has to design the MCLS architecture. There are three types of MCLS architectures, namely—static parallel (SP); multi-stage (MS) design; and three dynamic classifier selection (DCS)^59–61^.

One of the most famous MCLS architectures is Static Parallel by Zhu et al.^62^. For SP, two or more classifiers are developed independently and executed in parallel. The outputs generated by all base classifiers are then combined to determine a final classification decision (selected from a set of possible class labels). Many combination functions are available for this architecture, including majority voting, weighted majority voting, the product or sum of model outputs, the minimum rule, the maximum rule and Bayesian methods. Averaging is mainly used for regression problems, while voting is used for classification problems. There are two categories of SP-related MCLS: a single ML algorithm is used as base learning (homogenous parallel), and multiple ML algorithms are used as base learning (heterogeneous parallel). For the paper, the former category has been used.

The second type of MCLS architecture is MS design, where the classifiers (usually with no overlaps) are organised into multiple groups and then iteratively constructed in stages. At each iteration, the parameter estimation process depends on the classification properties of the classifiers from previous stages. As with SP, this design benefits from processing inputs in parallel. It ensures that labels are assigned using only the necessary features. In addition, the number and composition of stages used by the model have proven to have a significant impact on overall performance. Some MS approaches have been used to generate models applied in parallel using the same combination rules used for SP methods. For example, most boosting strategies have been shown to create weak classifiers, but they tend to form stronger ones^63^.

A dynamic classifier selection (DCS) is an ensemble learning architecture developed and applied to different regions within the problem domain. The technique involves training MCLS on the dataset and selecting the best prediction models. The *k*-NN approach is sometimes used to determine instances that are closely related to the unknown instance to be predicted (see Sect. “Single-classifier learning systems”). While one classifier may be shown to outperform all others based on global performance measures, it may not necessarily dominate all other classifiers entirely. Weaker competitors will sometimes beat the best across some regions (Kittler^64^). Research has shown DCS performs better than single classifiers and even better than combining all the base classifiers. Furthermore, Kuncheva^65^ approached DCS problems from a global and local accuracy perspective with promising results.

Ensemble learning of classifiers can be classified into three stages: (1) generation, (2) selection, and (3) integration. The objective of the first stage is to obtain a pool of models, followed by a selection of a single classifier or a subset of the best classifiers. Finally, the base models are combined to obtain the prediction for new or unknown instances. The aspect of multiple classifier systems is determining the number of component classifiers in the final ensemble (also known as ensemble size or cardinality) is the most important. The impact of ensemble size on efficiency in time and memory and predictive performance makes its determination a critical problem^65–68^, Li et al.^69^).

Furthermore, one should assume that diversity among component classifiers should be another influential factor in an accurate ensemble. However, no explanatory theory reveals how and why diversity among components contributes to overall ensemble accuracy. Therefore, all possible ensemble sizes and their respective diversities are considered for this paper.

Recently, Multi-Classifier-Based Boosting was introduced, where clustering and classifier training are performed jointly^70,71^. These methods have been applied to object detection, where the entire training set is available from the beginning. Other related works include multiple instance learning^72,73^ and multiple deep learning architectures^74^. The former algorithm learns with bags of examples, which only need to contain at least one positive example in the positive case. Thus, the training data does not have to be aligned. Mellema et al.^74^ developed the system using anatomical and functional features to diagnose a subject as autistic or healthy.

Ensemble methods offer several advantages over single models, such as improved accuracy and performance, especially for complex and noisy problems. They can also reduce the risk of overfitting and underfitting by balancing the trade-off between bias and variance, and by using different subsets and features of the data. Furthermore, they can provide more confidence and reliability by measuring the diversity and agreement of the base models, and by providing confidence intervals and error estimates for the predictions. Despite their pros, ensemble methods have some drawbacks and challenges such as being computationally expensive and time-consuming due to the need for training and storing multiple models, and combining their outputs. Additionally, they can be difficult to interpret and explain, as they involve multiple layers of abstraction and aggregation, which can obscure the logic and reasoning behind the predictions.

## Experiments

### Experimental set-up

The main aim of this randomized controlled experiment is to evaluate the effectiveness of five machine learning algorithms for predicting ASDT for a robot-assisted intervention group of autistic children and control receiving intervention by a human only. We further investigate how ASDT predictive accuracy could be improved using ensemble learning.

The investigations are conducted using a dataset of behavioural data and robot-enhanced therapy recorded from 61 children diagnosed with ASD^75^. The dataset covers 3000 therapy sessions and more than 300 h of treatment. Half of the children interacted with the social robot supervised by a therapist, while the other half was used as a control group (i.e. interacting directly with the therapist). In other words, the class attribute is the type of intervention – social robot-enhanced therapy condition (i.e. the interaction of an autistic child with a social robot) or social human therapy condition (i.e. the interaction of an autistic child with a human). The attributes are as follows:

1. The ability of the child to wait for his or her turn;
2. Social interaction and communication outcomes (engagement, eye contact, and verbal utterances);
3. Behavioural outcomes (stereotype behaviours, maladaptive behaviours, and adaptive behaviours); and
4. Emotional outcomes (functional and dysfunctional negative emotions, and positive emotions).

Furthermore, both groups followed the applied behaviour analysis (ABA) protocol. ABA uses scientific observations and principles of behaviour to improve and change behaviours of social interest^76^. In both the RET and SHT sessions, the children participated in a randomised manner to avoid ordering effects^32^. Participants in both groups went through a protocol of initial assessment, eight interventions, and a final assessment. The effect of the treatment was assessed using the Autism Diagnostic Observation Schedule (ADOS), in terms of the difference between the initial and final assessments^77^.

All therapy sessions were recorded using the same sensorized therapy (Fang et al.^78^). Each session was recorded with three red–green–blue (RGB) cameras and two Red–Green–Blue-Depth (RGBD) Kinect cameras, providing detailed information on children’s behaviour during therapy; the dataset comprises body motion, head position and orientation, and eye gaze variables, all specified as 3D data in a joint frame of reference. Other metadata attributes include participant age, participant gender numeric ID, target ability or task, therapy condition (response elaboration training or substitutive hormonal therapy) and date of therapy. A complete list of sensor primitives and associated methods is provided in Table 1.

**Table 1:** Sensor primitives extracted by the sensorized intervention table.

Sensor primitive	Interpretation method
Relative eye-gaze	Two-eye model-based gaze estimation based on RGBD^79^
Head pose	Pose from orthography and scaling with iterations (POSIT)^80^
Gaze estimation	A 3D gaze vector is achieved by combining the relative eye gaze with the calculated head pose (Fang et al.^78^)
Face detection	Boosted cascade face detector^81^
Facial features	Supervised descent method proposed by^82^
Face expressions	Frontalised local binary patterns (LBP) are classified using SVM^83^
3D skeleton	Microsoft kinect SDK
Action recognition	3D joints moving trend method based on skeleton data^84,85^
Object tracking	GM-PHD tracker^86^
Sound direction	Microsoft kinect SDK

This public release of the dataset does not include any footage of children. Instead, processed features of the recorded data are provided. According to the source of the data, informed consent was obtained from all subjects and/or their legal guardian(s) when the data was collected. The experimental protocols were approved by the University of Sk**ö**vde in Sweden which is the main source of the data. For more information, the reader can contact Billing et al.^75^.

In addition, metadata including participant’s ID, age, gender, and ASD diagnosis variables (3D skeleton comprising joint positions of the upper body; 3D head position and orientation; 3D eye gaze vectors; therapy condition; therapy task including joint attention, imitation and turn-taking; data and time of recording and initial ADOS scores) are included. As this was secondary data, no ethics committee had to approve the study within our environment. Furthermore, all methods were carried out following relevant guidelines and regulations.

For the simulations, five base classifiers were modelled using default hyper-parameters for each respective classifier. Each approach utilises a different form of parametric estimation or learning. For example, they generate various forms in linear models, density estimation, trees, and networks. These classifiers are among the top 10 most influential and popular algorithms in data mining^87^. They are all practically applicable to ASD, with known examples of their application within the robotics-enhanced therapy industry.

First, each state-of-the-art classification method (base classifier) was constructed using MATrix LABoratory or MATLAB software^88,89^. These base classifiers were later used and assessed as a benchmark against various MCL systems. It was evident that the benefits of using ensembles could not be achieved by simply copying an individual model and combining the individual predictions. For this reason, all possible combinations of the number of classifiers per ensemble were explored (i.e. from two classifiers per ensemble to five classifiers). These ensembles are defined as Multiple Classifier Learning Systems 2 (MCL 2) (for two classifiers per ensemble),Multiple Classifier Learning Systems 3 (MCL 3) (for three classifiers per ensemble); Multiple Classifier Learning Systems (MCL 4) (for four classifiers per ensemble), and Multiple Classifier Learning systems 5 (MCL 5) (for all five classifiers in the ensemble).

To assess the performance of the base classifiers, the training set—validation set—test set methodology is employed. First, the dataset was split randomly into a 60% training set for each run, a 30% validation set and a 10% testing set. To test the effectiveness of the classifiers, the dataset was further split randomly into 5-folds. The smoothed error rate (i.e. smoothing the normal error count using estimates of posterior probabilities and the posterior probabilities using Bayesian estimation with conjugate priors) was used as a performance measure in all the experiments. This rate was used primarily for its variance reduction benefit and for dealing effectively with a tie between two competing classes^90^. The F-measure (score) was also used as a performance measure for the single classifier empirical comparison experiments. The benefit of the F-measure is that it considers the models’ ability over two class attributes, which makes it a robust gauge of model performance.

Feature (factor) ranking and selection methods have been implemented with two basic steps of a general architecture for our experiments: subset generation and subset evaluation for the ranking of each feature in every dataset. Then, the filter method is used to evaluate each subset. Overall, a mutual information-based approach on the single classifier that exhibits the lowest error rate is utilised for this task. Mutual information calculates the reduction in entropy from the transformation of a dataset. The technique is summarised below.

A DT classifier has implicit feature selection during the model-building process. It identifies and ranks the features (factors) that significantly impact or contribute to ASD. The set of features available forms the input to the algorithm with a DT as output. The purpose of this technique was to discard irrelevant or redundant features (factors) from a given vector. For the paper, feature (factor) selection was used by evaluating the mutual information gain of each variable in the context of the target variable (robot-child against human-child therapy).

The fixed-effect model (Kirk^91^) is used to test for statistical significance of the main effects (i.e. the five single classifiers; twenty-three multiple classifier systems, three multiple classifier architectures and five resampling procedures) versus their respective interactions. Each experiment is randomly replicated five times (5-fold) making it a total of 5 × 23 × 3 × 5 × 5 = 8625 experiments.

### Experimental results

Experimental results on the ASDT predictive performance of single classifiers (on the one hand) and MCLS (on the other hand) are described. The behaviour of multiple classifiers is explored for different MCLS architectures and resampling procedures.

The results are presented in three parts.

The first part compares the performance and robustness of five single-classifier learning systems in predicting ASDT in autistic children. The second part investigates the performance of MCLS (i.e. ensembles, resampling procedures, and architectures) to determine if there is an improvement in ASD therapy predictive accuracy. These overall results are for each MCL system. They are averaged for all ensemble learning combinations about resampling procedures and architectures. Then, the experimental comparison of MCL systems (for all possible ensemble combinations) is presented. Finally, the behavioural factors (in ranking order) that contribute to and are critical when addressing the ASD problem have been identified.

**Figure 1:**
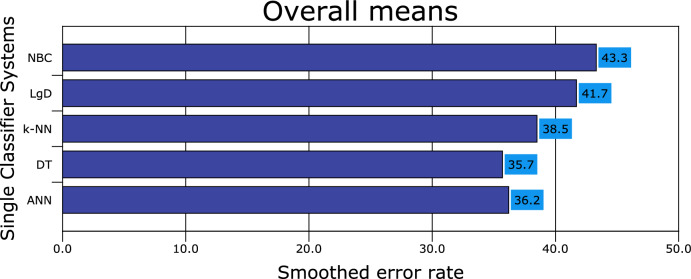
Single classifier systems.

**Figure 2:**
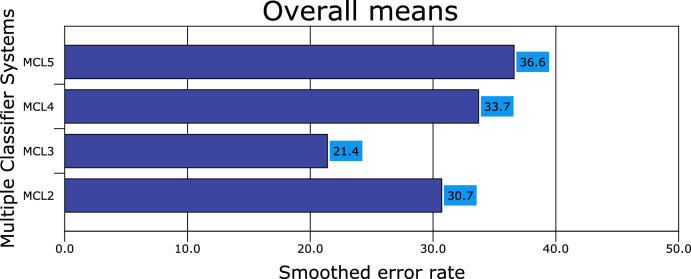
Multiple classifier systems.

**Figure 3:**
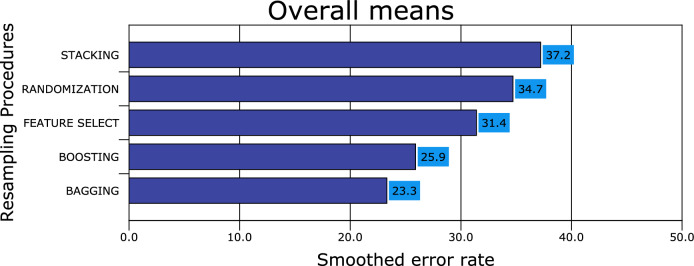
Resampling procedures.

**Figure 4:**
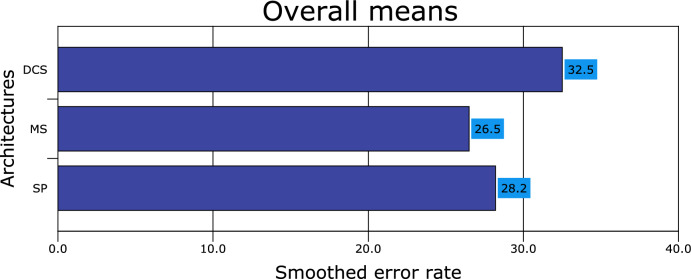
Multiple classifier systems architectures.

**Figure 5:**
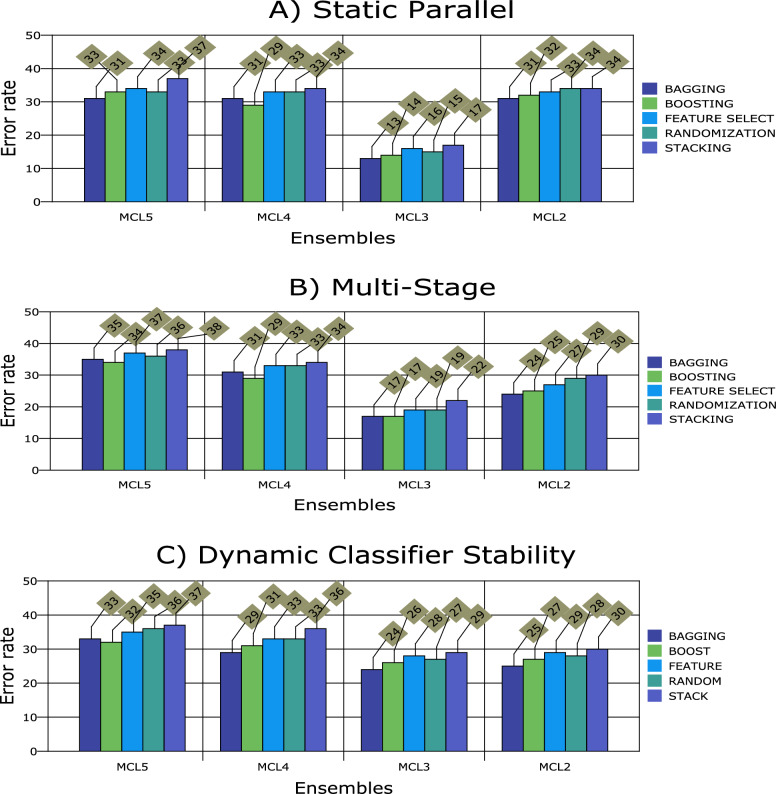
Multiple classifier learning systems (overall results).

**Figure 6:**
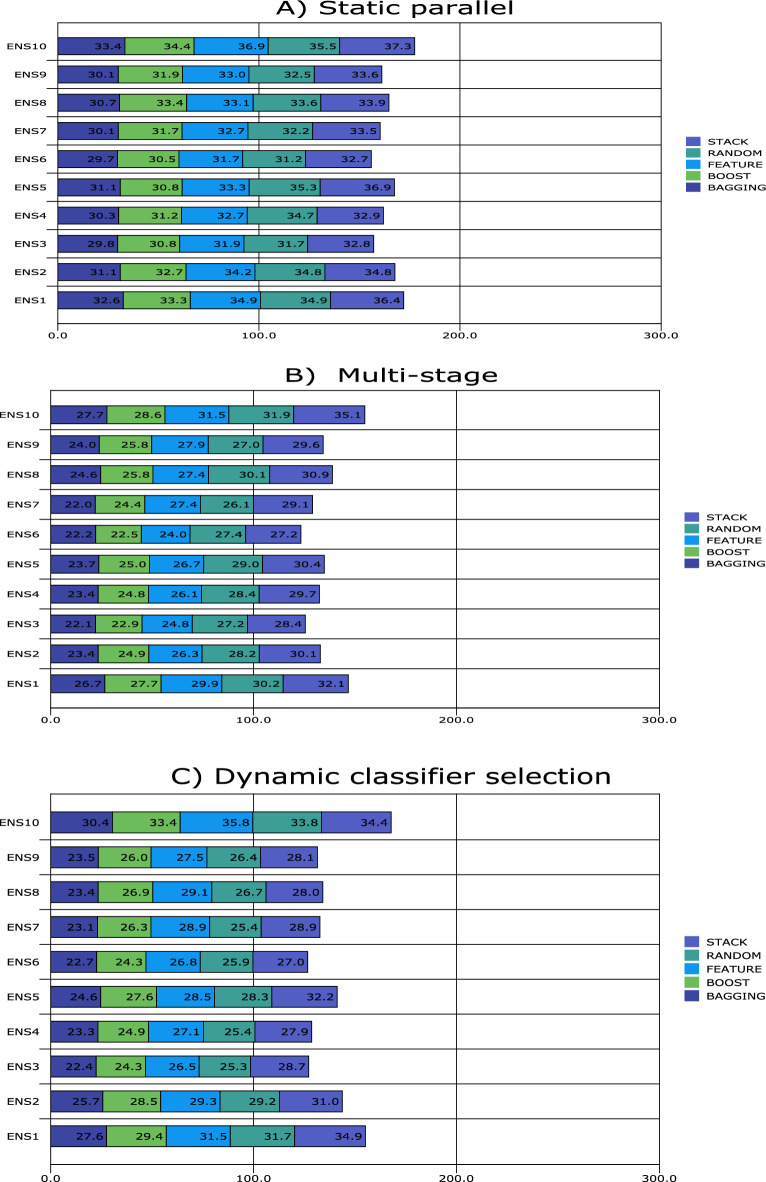
Multiple classifier learning systems **2**.

**Figure 7:**
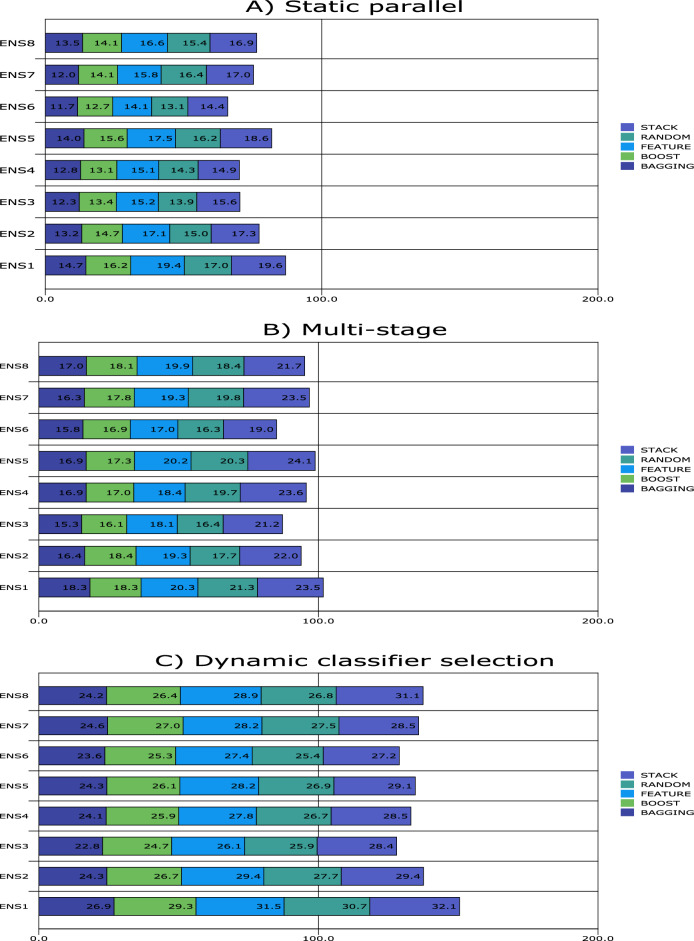
Multiple classifier learning systems **3**.

**Figure 8:**
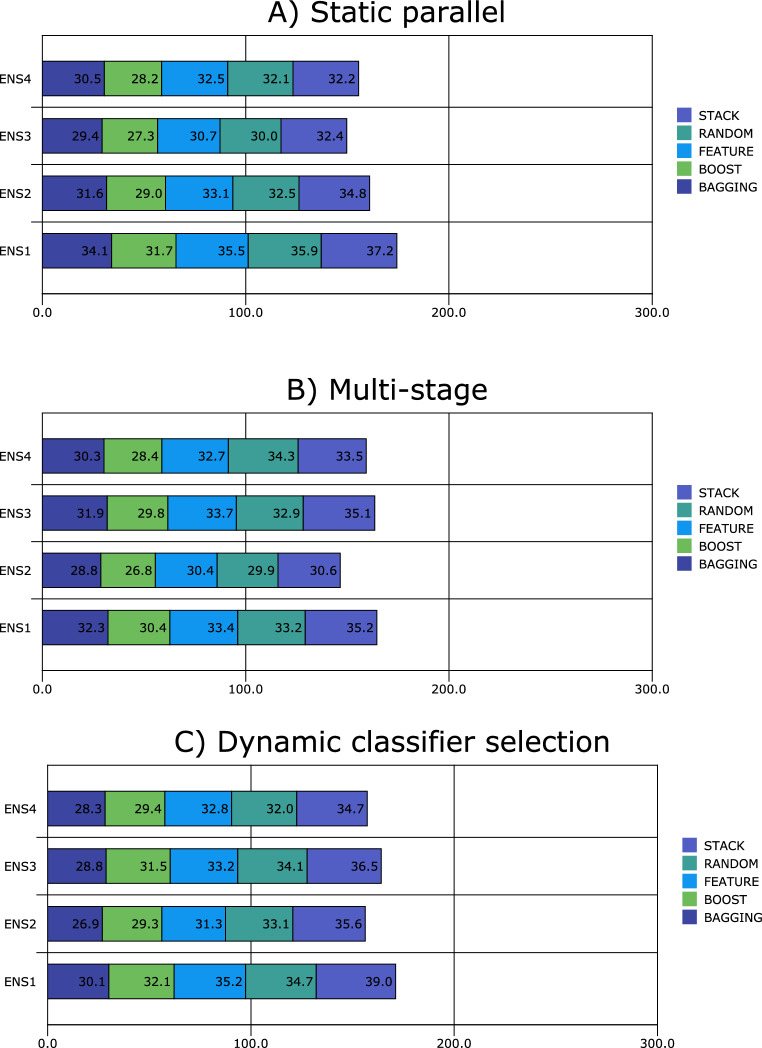
Multiple classifier learning system **4**.

**Figure 9:**
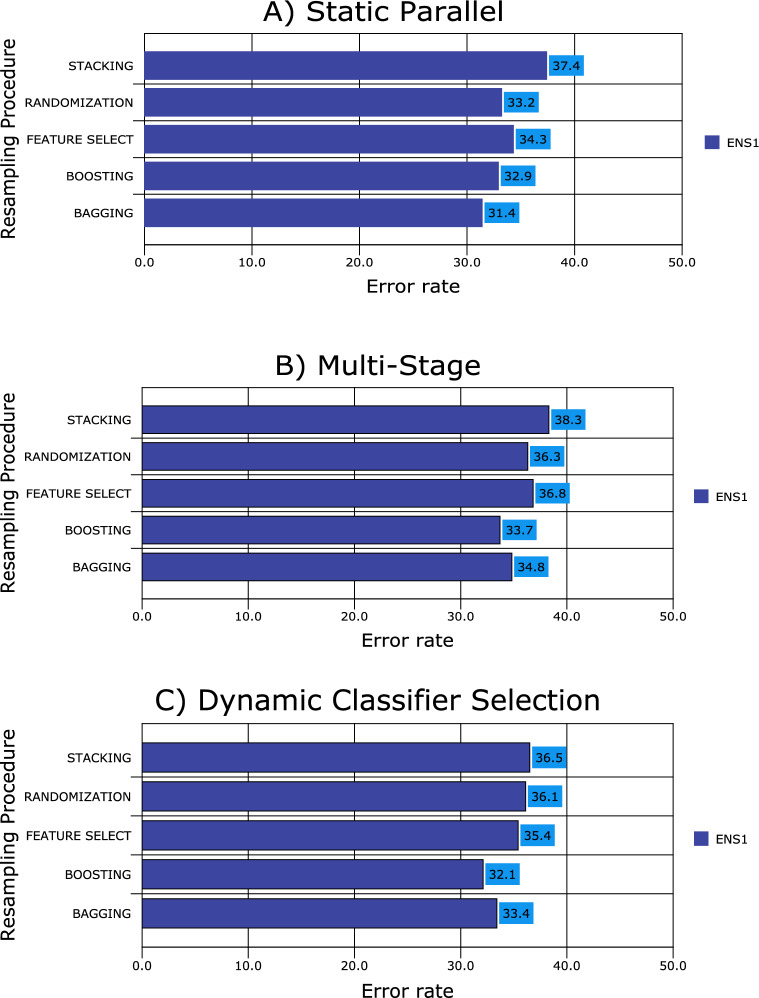
Multiple classifier learning systems **5**.

Figures 1, 2, 3, 4, 5, 6, 7, 8 and 9 plot the smoothed error of the instances learned on the target domain, averaged over five-fold cross-validation runs by each one of the methods. The same folds were used to evaluate each method. All the main effects (i.e. base or single classifier systems, MCL systems, resampling procedures, and MCL systems’ architectures) were significant at the 5% level, with F-ratios of 131.7, 71.4, 513.6 1132.6, respectively.

From Fig. 1, it follows that DT is the best base classifier, exhibiting a smoothed error rate of 35.7% ± 1.7% (or 0.643 accuracies; F-measure of 0.631). The second-best base classifier is ANN, followed by *k*-NN and LgD with smoothed error rates of 36.2% ± 2.2% (0.625 accuracies; F-measure 0.6035), 38.5% ± 1.6% (0.618 accuracies; F-measure 0.603), and 41.7% ± 1.9% (0.583 accuracies; F-measure 0.575). Finally, the worst performance is by the NBC with a smooth error rate increase of 43.3% ± 1.4% (0.567 accuracies; F-measure 0.531). The most relevant attributes to predicting ASD therapy are communication (non-verbal), eye contact and social interaction for the single classifiers.

From Fig. 2, the performance of the single baseline model is taken as the reference point. It appears that the performance of all the MCL systems is statistically significant at the 95% confidence level compared to single-classifier learning systems. The MCL systems when the ensemble size is composed of only three classifiers achieve the least smoothed error rate of 21.4% ± 1.9%. All ensembles with only two classifiers exhibit the second-best performance (a smoothed error rate of 30.7% ± 1.8%), while those with only four classifiers take the third spot. The worst performance is when the ensemble comprises all five classifiers (with a smoothed error rate of 36.5% ± 2.2%). The difference in performances between the four ensembles is statistically significant at the 5% significance level. Eye contact and social interaction were the most relevant features when predicting ASD (using multiple classifier systems).

All ensemble classifiers with bagging achieve the lowest error rate (23.3% ± 1.9%), followed by boosting (25.9% ± 1.5%), feature selection (31.4% ± 1.7%) and randomization (34.7% ± 1.5%), respectively. Stacking ensemble classifiers achieve the lowest accuracy rate (37.2% ± 2.1%). From the accuracy point of view, the performance differences of the ensemble classifiers were statistically significant with a 0.95 degree of confidence (Fig. 3).

From Fig. 4, it appears that all the multiple classifier systems have a more significant robust effect when the multi-stage design is used as an architecture (accuracy rate of 73.5% ± 1.8%), followed by static-parallel and dynamic classifier selection with accuracy rates of 71.8% ± 1.3% and 67.5% ± 1.7%, respectively. The difference in performance between the architectures was significant at the 5% level.

The results presented in Fig. 5 show all the MCLS performing worse under the dynamic classifier selection (an error rate of 32.5% ± 1.7%) compared with single parallel and multi-stage. On the other hand, the MCLS performs slightly better when the multi-stage architecture design is used (26.5% ± 1.5%) than a single parallel (28.2% ± 2.3%). Thus, the difference in performance between the three architectures was significant at the 5% significance level (following a similar pattern to Fig. 4 results).

The three-way interaction effect between multiple classifier learning systems, architectures and resampling procedures was found to be statistically significant at the 5% level. This means that the interaction between two attributes is different across the levels of the third attribute. In other words, there was a two-way interaction between resampling methods and multiple classifier learning systems varying across architectures; a two-way interaction between architectures and resampling methods varying across multiple classifier learning systems; and a two-way interaction between architectures and multiple classifier learning systems varying across resampling methods. The results are summarised in Fig. 5.

It follows that all MCLS perform differently from each other when predicting ASD therapy, with significant error rate increases observed for ensembles with five or four classifiers compared to those with three or two classifiers per ensemble. Ensembles of three single classifiers achieve the highest accuracy rates with ensembles of five single classifiers achieving the lowest accuracies. Ensembling with boosting outperforms the other resampling methods with ensembling with stacking achieving the lowest accuracy rate. This is the case across all three architectures.

Static parallel multiple classifier learning for ASD therapy prediction achieves the highest accuracy, followed by multi-stage and dynamic classifier stability multiple classifier systems, respectively. Once again ensembling with bagging achieves the highest accuracy rates with poor performance for ensembling with stacking. This is the case across all multiple classifier learning systems.

The performance of all multiple classifier systems in terms of predicting ASDT is significantly different across all three architectures. Major differences are noticeable for multi-stage design against single parallel, with minor differences observed for multi-stage design against dynamic classifier selection. Once again, the results show that MCLS built through bagging is the best technique for predicting ASDT, followed by boosting, feature selection, randomisation and stacking, respectively.

From Fig. 6A, the effect of the resampling procedures multiple classifier learning system 2 (MCLS 2) is transparent. MCLS 2 exhibits the worst performance for stacking, closely followed by feature selection and randomisation. The best overall performance for a static parallel architecture comes about when bagging is used. In contrast, the best performance is observed when decision trees and logistic discrimination are the two components of the ensemble. The ensembles of artificial neural networks and decision trees and logistic discrimination and naïve Bayes classifiers exhibit the worst performances.

From Fig. 6B, bagging exhibits minor error rate increases (with tight competition from boosting) for MCLS 2 and when the multi-stage architecture is used. One striking outcome is the artificial neural networks and logistic discrimination ensemble performance, which compares favourably with a Decision tree and logistic discrimination ensemble. However, the ensembles with artificial neural networks decision trees and logistic discrimination and naïve Bayes classifiers exhibit one of the worst performances for MCLS 2. Another poor performance is when the *k*-nearest neighbour and naïve Bayes classifiers are used as ensemble components, primarily when randomisation is used.

The dynamic classifier selection system is observed. At the same time, stacking continues to struggle and achieves the worst performance, especially when the artificial neural network and a decision tree (on the one hand) and logistic discrimination and naïve Bayes classifier (on the other hand) are components of the ensemble (Fig. 6C). The best-performing ensembles are artificial neural network and logistic discrimination, and the decision tree and logistic discrimination are components.

The performance of methods for multiple classifier learning (MCL3) follows a similar pattern to the one observed for MCL2 (Fig. 7).

Figure 7A also shows smaller increases in error rates for all the resampling procedures for static parallel than the same architecture for MCL2. The best-performing ensemble is when Decision Trees, *k*-nearest neighbour and logistic discrimination are components. On the other hand, poor performances are observed when the Artificial Neural Network, Decision Trees k-nearest neighbour and Artificial Neural Network, and *k*-nearest neighbour and naïve Byes classifiers are components of the ensemble. This is the case for the feature selection and stacking resampling procedures.

The methods for multi-stage design (Fig. 7B) are nearly identical to those observed for MCLS2, with all ensembles achieving higher accuracy rates when bagging and boosting are used. Otherwise, on average, the performance of all the methods worsens when stacking is used. The best-performing ensemble is the decision tree, *k*-nearest neighbour, and logistic discrimination (primarily feature selection, randomisation and stacking). For stacking, the ensemble method composed of an Artificial Neural Network, *k*-nearest neighbour, and naïve Bayes classifiers proves to be the worst-performing.

The impact of MCLS 3 on predictive accuracy is shown in Fig. 7C. Once again, bagging yields the best performance, closely followed by boosting with severe competition from randomisation. Once again, the best-performing ensemble is when the artificial neural network, decision trees and naïve Bayes classifiers are components. The ensemble with the *k*-nearest neighbour, logistic discrimination and naïve Bayes classifier drops from being the third-best performing (when stacking and multi-stage design is used) to being one of the worst (when stacking and dynamic classifier election are used).

Overall, all the MCLS 3 systems perform better when static parallel is used, followed by multi-stage and dynamic classifier selection.

Figure 8A follows that when using static parallel to build an MCLS 4, boosting is the best technique for dealing with the ASD spectrum disorder problem, with an Artificial Neural Network, *k*-nearest neighbour, logistic discrimination, and naïve Bayes classifiers as components for the ensemble. On the other hand, the ensemble with an artificial neural network, decision trees, *k*-nearest neighbour and logistic discrimination as components achieves the worst performance. This is the case at all resampling procedure levels (i.e. bagging, boosting, feature selection, randomisation and stacking).

It follows from Fig. 8B that the best technique for handling ASD Spectrum Disorder for a multi-stage design and across various resampling procedures is boosting, closely followed by bagging. However, poor performances are observed for feature selection, randomisation, and stacking methods. Also, the ensemble with an artificial neural network, decision trees, *k*-nearest neighbour and logistic 0 discrimination as components exhibit the worst performance.

For MCLS 4, bagging using dynamic classifier selection shows superior performances to the other resampling procedures (Fig. 8C). The best-performing ensemble (across bagging, boosting, and feature selection) is where components of artificial neural network, decision trees, k-nearest neighbour and naïve Bayes. On the other hand, randomisation and stacking of an ensemble with an Artificial Neural Network, k-nearest neighbour, logistic discrimination, and naïve Bayes perform best.

For this kind of problem, it seems that building an MCLS 5 using a static parallel architecture performs better compared with other architecture such as dynamic classifier selection and multi-stage design (Fig. 9A–C). Additionally, ensembling learning with boosting appears to be more effective especially when dynamic classifier selection and multi-stage design are used while bagging appears to be more effective when static parallel is used, outperforming resampling methods like feature selection, randomisation and stacking in some situations. Another good performance is when static parallel and multi-stage design ensemble learning is used with randomisation. Overall, ensemble learning with stacking is the worst-performing method across all three architectures.

Social difficulties are a core of ASD with one of the many psychological factors being the lack or low levels of joint attention with the interaction partners. Given the attention the use of social robots has received in ASD interventions, it was important to investigate the most significant attributes that contribute to ASD therapy (i.e. RAAT vs. SHT) and rank them accordingly. Such ranking will help investigate if RET produces similar patterns in comparison with SHT.

Feature selection is one of several ranking approaches that have been used for dealing with the high dimensionality of data and improving classification accuracy^92^. One of the goals of feature selection in machine learning is to find the best features to build applicable models of a studied phenomenon (for example, removing non-informative or redundant ASD predictors from the model). There are many feature selection algorithms including filtering, encapsulation and embedded ones (Tang and Pen^93–95^. Many feature selection techniques are classified into supervised (wrapper filter, intrinsic, embedded) and unsupervised learning (for unlabelled data).

**Figure 10:**
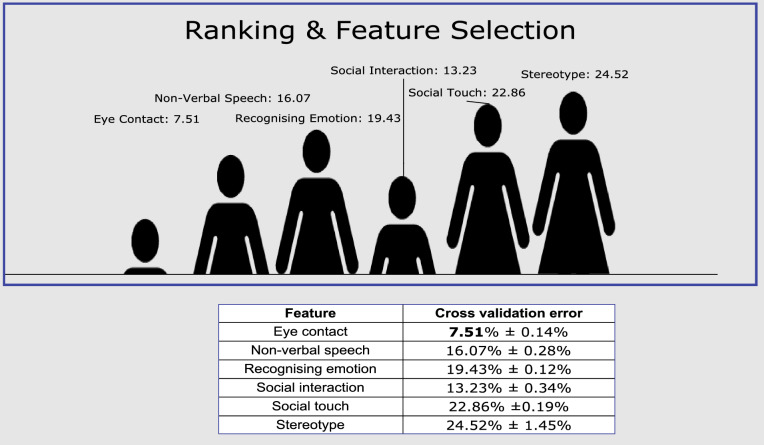
ASD therapy features sorted by relevance.

The goal of feature selection techniques in artificial intelligence is to find the best set of features that allows one to build optimised models of a studied phenomenon (ASDT in this case). There are many feature selection algorithms but for this paper, we use the classic methods for constructing a decision tree which is the same process of feature selection. The decision tree algorithm (a supervised learning and embedded approach) was used to select the features in ranking order and according to the mutual information criterion whereby node impurities in the decision tree are utilised. The strengths of the decision tree algorithm are high classification accuracy and strong robustness. The feature selection process algorithm results modelled to obtain features considered most relevant to ASD therapy enhancement and their merit value ranks each feature are analysed and summarised in Fig. 10.

In terms of ranking, the results show *eye contact* yielding the slightest cross-validation error ($7.51\%\pm 0.14\%$) followed by *social interaction* ($13.23\%\pm 0.34\%$) and *non-verbal speech* ($16.07\%\pm 0.28\%$), respectively. Otherwise, *social touch* and *stereotype* are the two features exhibiting error rates of more than 20%, i.e. ($22.86\%\pm 0.19\%$) and ($24.52\%\pm 1.45\%$), respectively. In addition, all the features were significantly different at the 5% level of significance. In other words, eye contact for autistic children appears to have more impact on ASD-enhanced therapy compared to, say, stereotypes or social touch.

## Remarks and conclusion

In this paper, novel research is performed regarding the exploration and prediction intervention use in autistic children using ASD-specific characteristics. Open questions related to predicting with confidence addressed include: How can ASDT data be utilised effectively to achieve more efficient confidence-based predictions using ensemble classifiers? To this end, the significant contributions of the paper include showing the robustness of single classifiers for predicting ASDT enhancement using a social robot against a human (therapist). Additionally, it shows how MCLS provides therapy enhancement performance improvements over the single base classifier (including the best-performing one). Further, a tree-based approach is used to quantitatively determine the importance of each physical attribute (according to mutual information-based ranking). Additionally, the conclusions are that single training classifiers can obtain influential ensembles in several different ways. Still, that high average individual accuracy or much diversity would generate influential ensembles.

There are several notable takeaways from this work.

First, ensembles are built with a combination of three classifiers and using bagging to achieve the perfect fit. The good performance of these ensembles could be attributed to the stable nature of nearest neighbour and linear threshold algorithms when they were core components of the ensemble. Ensembles built with dynamic classifier selection by segmenting the population into several sub-regions consistently perform poorly. However, the performance of most static parallel and multi-stage combination ensemble strategies provides statistically significant improvements over the single best classifier. We understand that in very large datasets, randomisation is expected to do better than, say, bagging or boosting but given the size of the ASDT data, bagging achieved the best results.

Eye contact and interactive communication appear to be the critical behavioural factors to be considered when dealing with ASD therapy. However, this can be argued because of the inability of children with this disorder to communicate and use language, which depends heavily on their intellectual and social development. In other words, some children with ASD may not be able to communicate using speech or language, and some may have minimal speaking skills. Therefore, joint attention in children could be another factor that needs consideration when dealing with ASDT.

Previous studies did not provide a clear conclusion about the predictive accuracy of multiple classifier systems for intervention use in autistic children. This study was the first of its kind focusing on predictive intervention use of ASDT using single classifiers and multiple classifier systems. When creating confidence-based predictors using conformal prediction, several open questions regarding how knowledge can be extracted from data using ensemble learning. The study has utility for researchers, clinicians and parents alike. It affords the potential to learn and become socially fluent no matter how strong the autism impairments may be. Although a cure for ASD has not been found yet, accurate prediction of ASDT could lead to improved outcomes or even a complete cure. Additionally, the study paves the way for investigating if an Artificial Intelligence device could be programmed to notice and react to verbal and non-verbal responses. These could include facial expressions, body movements, and vocal and physiologic reactions from an autistic child (i.e. could artificial intelligence replace a therapist?). This assertion is based on our study where the application of AI for ASDT prediction shows promising results.

The study is based on children over the age of 3 years. In a subsequent study, the datasets of children of all ages will be critically analysed to train the therapeutic prediction model. The focus will be to collect more data from various sources and age groups and further improve the proposed ML classifier to enhance its accuracy. Furthermore, a more state-of-the-art classification method (including single classifiers like support vector machines that were not considered in these experiments) will also be considered. The focus of the study was on children with ASD, our next research will be on both autistic children against autistic adults. Additionally, our study was purely focused on behaviours. Future work will investigate specific cognitive mechanisms that might be targeted or affected by robot vs. human interactions.

In sum, this research provides an effective and efficient approach to predicting and detecting ASD traits for children above three years. This is because tests and diagnoses of ASD traits are costly and lengthy. The difficulty of detecting ASD in children and adolescents does not help another cause for the delay in diagnosis. Thus, with the help of accurate ASD spectrum disorder predictive accuracy, an individual can be guided early to prevent the situation from getting any worse and reduce costs associated with such delay.

## Data Availability

Many thanks are extended to the Swedish Data National Service for making the dataset available. The data that support the findings of this study are available from the University of Skövde, Skövde, Sweden but restrictions apply to the availability of these data, which were used under license for the current study, and so are not publicly available. Data are however available from the authors upon reasonable request and with permission of the University of Skövde, Skövde, Sweden. [Contact: Prof Erik Billing < erik.billing@his.se > . There are no biomedical financial conflicts of interest to disclose. Also, this research received no specific grant from any funding agency in the public, commercial, or not-for-profit sectors.
